# Mechanisms of Chronic Metabolic Stress in Arrhythmias

**DOI:** 10.3390/antiox9101012

**Published:** 2020-10-19

**Authors:** Blake H. Gowen, Michael V. Reyes, Leroy C. Joseph, John P. Morrow

**Affiliations:** Department of Medicine, College of Physicians and Surgeons of Columbia University, New York, NY 10032, USA; bg2691@cumc.columbia.edu (B.H.G.); michaelvreyes91@gmail.com (M.V.R.); lcj2117@cumc.columbia.edu (L.C.J.)

**Keywords:** cardiac arrhythmia, myocardium, high-fat diet (HFD), metabolism, obesity, diabetes, mitochondria, antioxidants

## Abstract

Cardiac arrhythmias are responsible for many cardiovascular disease-related deaths worldwide. While arrhythmia pathogenesis is complex, there is increasing evidence for metabolic causes. Obesity, diabetes, and chronically consuming high-fat foods significantly increase the likelihood of developing arrhythmias. Although these correlations are well established, mechanistic explanations connecting a high-fat diet (HFD) to arrhythmogenesis are incomplete, although oxidative stress appears to be critical. This review investigates the metabolic changes that occur in obesity and after HFD. Potential therapies to prevent or treat arrhythmias are discussed, including antioxidants.

## 1. Introduction

Cardiovascular disease (CVD), including coronary heart disease, heart failure, stroke, and hypertension, persists as the leading cause of death worldwide despite advances in medical treatment [1,2,3,4]. In the U.S.A., elevated rates of CVD are caused by an increasing prevalence of risk factors including obesity, diabetes, and the consumption of foods high in fat and/or sugar [4,5,6,7]. Arrhythmias are a common form of heart disease, especially in older populations. Atrial fibrillation is one of the most common chronic arrhythmias and is more frequent in obese people. Sudden cardiac death (SCD) is responsible for many CVD-related deaths and is mostly caused by ventricular arrhythmias [1,8]. Although the likelihood of arrhythmic events increases with age, there has been an increase in prevalence of arrhythmias across all demographics [4]. Risk factors for arrhythmias include metabolic factors such as obesity, diabetes, and consuming foods high in saturated fat, though these groups overlap and distinctions between these groups have not always been well defined in the literature.

Public health concerns regarding the surge in obesity (defined in humans as having a body mass index, or BMI, of 30.0kg/m^2^ or more) over the past few decades, concerning trends in dietary patterns, and the increased prevalence of CVD and arrhythmias motivate a closer examination of the metabolic impacts of a chronic high-fat diet (HFD), both concurrent with and independent of comorbidities such as obesity. With a global increase in the consumption of high-fat foods and prevalence of obesity, antioxidant therapy to combat these metabolic conditions has potential for cardioprotection. The aim of this review is to examine the potential mechanisms that mediate the relationship between obesity or the long-term consumption of high-fat foods and the development of pro-arrhythmogenic metabolic conditions, as well as novel antioxidant approaches to pharmacological treatment. For a more clinically oriented review, the interested reader is referred to this recent publication: [9].

## 2. Basics of Myocardial Metabolism

Since the presence of metabolism can be used to define what is alive (and in this sense viruses are not alive despite being composed of proteins and nucleic acids), it is trivial to state “metabolism is involved in disease X.” Of course, it is. How could metabolism not be involved? Nonetheless, we accept that some diseases are more metabolic than others and figuring out the detailed mechanisms is not a trivial undertaking. Given the intense metabolic demands on the heart, abnormal metabolism is important for heart disease as a broad category.

In order to understand myocardial metabolism, we begin with a brief review of mitochondria. Mitochondria account for approximately one-third of cardiac myocytes’ cell volume—the most densely packed mitochondria of any tissue [10]. Approximately 95% of all ATP used by cardiac myocytes is derived from mitochondrial oxidative phosphorylation (OXPHOS) as opposed to glycolytic metabolism [10,11,12]. As the heart develops, cardiac mitochondria form mitochondrial networks via fission and fusion, optimizing OXPHOS [13]. Mitophagy, the removal of dysfunctional mitochondria into lysosomes for degradation, provides additional quality control in regulating cardiac energetics [11].

β-oxidation of fatty acids provides a constant supply of energy to the heart since lipids contain more than twice the energy per gram compared to carbohydrates and protein. In fact, conditions such as heart failure (HF), arrhythmias, and ventricular hypertrophy are, in part, due to mitochondrial remodeling that results in reduced capacity for fatty acid oxidation (FAO) [11]. With such reliance on FAO, however, comes the risk of lipid-overload (also called lipotoxicity) in cases of hyperlipidemia or the long-term consumption of an HFD [14] In particular, excessive fat intake could result in oxidative stress from the overproduction of reactive oxygen species (ROS) including hydrogen peroxide and superoxide [13]. These molecules are responsible for cellular oxidative damage in the heart [13].

In order to protect itself against oxidative stress, the heart can express proteins that result in increased antioxidant activity, for example forkhead box O3a (FOXO3a), catalase, and metallothionein 2 (MT2) [15]. This may be initiated, in part, by the presence β-hydroxybutyrate (β-OHB), a ketone species upregulated in conditions of prolonged fasting and intense exercise [15]. β-OHB acts by inhibiting the activity of histone deacetylases 1 and 2 (HDAC-1,2), which otherwise function to suppress gene transcription by revoking access of transcriptional machinery [16]. However, individuals who regularly consume foods high in fat—and thus accumulate triacylglycerol (TAG) in their cardiac tissue—are unlikely to produce high enough levels of β-OHB for physiological relevance, and suppression of FOXO3a and MT2 will prevail. There is, however, recent evidence to suggest that the chronic consumption of an HFD results in the upregulation of enzymes responsible for ketone body synthesis from FAO [14]. This could provide a mechanism for reducing TAG pools in cardiac myocytes in the absence of fasting or exercise, though further investigation is needed. The heart (at least in mice) exhibits an additional mechanism akin to that of the liver and small intestine for removing excess lipid: The synthesis of apolipoprotein B (apoB) [17,18]. This lipoprotein allows for the regulation of TAG stores in cardiac myocytes by packaging and dispersing TAG into systemic circulation [17]. This suggests a potential mechanism for detoxifying the “fatty heart” following chronic HFD consumption.

Since the effects of saturated fat of cardiac myocytes seem like an important topic, one might think that there would be an extensive body of literature examining this, but, in fact, there are very few publications in this area. In a set of reductionist experiments, we exposed isolated cardiac myocytes from wild-type mice to physiologic levels of fatty acids [19]. The saturated fatty acid palmitate caused a decrease in mitochondrial respiration in cardiomyocytes, as quantified by a Seahorse system. We found that palmitate, but not the monounsaturated fatty acid oleate, caused an increase in total cellular ROS and mitochondrial ROS. Additional experiments demonstrated that palmitate depolarized the mitochondrial inner membrane and caused mitochondrial calcium overload by increasing sarcoplasmic reticulum calcium leak. Inhibitors of protein kinase C (PKC) or nicotinamide adenine dinucleotide phosphate (NADPH) oxidase 2 (NOX2) prevented mitochondrial dysfunction and the increase in ROS. NOX enzymes are an important source of ROS in myocytes. NOX2 has a physiologic role in regulating calcium release from the sarcoplasmic reticulum (SR) in cardiomyocytes [20]. NOX2 is known to be involved in the pathophysiology of several forms of heart disease, reviewed in [21]. Since NOX2 activation is required for amplification of palmitate-induced mitochondrial ROS in cardiomyocytes, there appears to be an interaction between NOX2 and mitochondrial ROS, an example of ROS-induced ROS release (RIRR). Cardiac mitochondria are physically in close proximity to the SR, forming microdomains [22]. This supports the scientific premise that increased SR calcium leak could lead to mitochondrial calcium overload and increased mitochondrial ROS could, in turn, worsen calcium handling abnormalities. However, experiments with isolated cardiomyocytes do not necessarily correspond directly to in vivo pathology. Additional work describing the role of NOX2 and arrhythmia in vivo is described in a later section.

## 3. Types of Dietary Fat

Though the term “high-fat diet” is commonly used in research publications without specifying the source of fat, it is important to consider the different effects of various classifications of fatty acids: Saturated, monounsaturated, polyunsaturated, and trans fats. Importantly, naturally occurring vitamins—such as vitamins A and E—are fat-soluble and can only be absorbed with fatty foods; thus having at least some form of fat in the diet is essential [23].

There is clinical evidence that consuming foods predominately containing polyunsaturated fatty acids (PUFAs) as the source of fat leads to cardioprotective and anti-inflammatory outcomes [24,25,26]. PUFAs can further be classified into ω-3 (e.g., α-linolenic acid) and ω-6 (e.g., linoleic acid) FAs, with ω-3 FAs being primarily responsible for the observed anti-inflammatory action of PUFAs [26]. Similar to PUFAs, monounsaturated fatty acids (MUFAs), such as oleic acid (the major component of olive oil), also result in better cardiovascular outcomes in comparison to saturated fats [26]. Consuming foods high in saturated fat increase risk of stroke, CVD, and SCD [23,27]. Trans fats, which are artificial and have been banned in several countries as food ingredients, further increase the risk of adverse cardiovascular events beyond that of saturated fat [28]. It is crucial to examine the precise fat content when evaluating the effects of an HFD with respect to the development of CVD and arrhythmias. There is some evidence that different types of dietary fat have differential effects on ion channels, which could relate to the risk of arrhythmia, reviewed in: [29]. Thus, when evaluating studies utilizing a high-fat diet, it is essential to consider the composition of the fat in the diets. With methodologies including diets spanning from 33% up to 80% kcal from fat, it is recommended that researchers report details on the dietary composition including both the percentage of fat as well as the type of fat used.

## 4. Cardiac Abnormalities Caused by Diet-Induced Obesity (DIO)

At present, people are significantly more likely to become obese and develop diabetes than people living 50 years ago, underscoring the importance of identifying protective therapies and lifestyle recommendations for an environment enriched with calorie-dense foods [30]. Obesity causes a variety of cardiac abnormalities in humans and laboratory animals [31]. While the oxidative stress consequences of long-term HFD consumption can act independently of obesity, individuals with obesity and/or diabetes are at an increased risk of developing CVD. In older humans, obesity is associated with great left-ventricular mass (estimated by echocardiography), indicating left ventricular hypertrophy [32]. The same study showed mild impairment of contractility and diastolic function in obese women compared to age-matched, normal-weight women; these parameters were not significantly different in obese men compared to age-matched, normal-weight men. Concerningly, left ventricular hypertrophy and diastolic dysfunction have also been detected in obese children and adolescents [33,34].

DIO rodents also have morphologic changes in that they develop cardiac hypertrophy [35,36]. The combination of DIO and pressure overload results in greater hypertrophy than either factor alone [37]. Some groups have reported that DIO or HFD rodents have a decrease in systolic function [36,38]. Other groups have not found a decrease in systolic function, usually measured by echocardiography, even after longer durations of HFD [39,40] These inconsistencies could be due to different research groups using different strains of mice and/or different forms of HFD. Mitochondrial ROS have been implicated in these structural abnormalities [41].

Obese humans have abnormal cardiac electrophysiology, most commonly measured by electrocardiography (ECG). Specifically, obese humans have increased frequency of ventricular ectopy (premature ventricular complexes: PVCs) and abnormal cardiac repolarization, measured as long QT (LQT) [42,43,44]. LQT is an independent risk factor for cardiovascular mortality [45,46]. Humans with obesity or diabetes also have increased repolarization dispersion, a measure of repolarization heterogeneity [47,48,49]. Abnormal repolarization can be a substrate for ventricular tachycardia and/or ventricular fibrillation, which can cause SCD. Obese humans do, in fact, have a significantly increased risk of SCD, compared to age-matched controls, a finding that has been seen in several studies from several different countries [8,50,51,52] While some of the increased risk of SCD is attributable to acute ischemia from atherosclerosis and thrombosis, there are data from autopsy studies supporting other mechanisms that can be involved [52]. This review will concentrate on ischemia-independent mechanisms of arrhythmogenesis.

Weight-loss surgery decreases both QT and QT dispersion, indicating that this is a causal effect rather than an association [53]. Obese humans also have atrial abnormalities, described in more detail below. Epidemiologic observations report a 5% increase in risk of atrial fibrillation (AF) per each unit increase in body mass index (BMI) [2,25,54]. There are data from humans and mouse models supporting the idea that increased heterogeneity of atrial repolarization contributes to the increased risk of AF [55].

Our prior work has shown that wild-type mice develop heart rhythm abnormalities when they are made obese by eating an HFD for about three months [35]. We were the first to show that DIO wild-type mice have heart rhythm abnormalities similar to obese humans, long QT and PVCs, validating this animal model. Protein kinase D (PKD), a serine/threonine kinase, which is related to calcium/calmodulin-dependent protein kinase (CaMK), is activated in the obese heart. PKD is known to cause cardiac hypertrophy from pressure overload [56] and also reduces the protein levels of the transcription factor cAMP response element-binding protein (CREB) [57]. Our work showed that CREB upregulates transcription of several voltage-gated potassium channel (Kv) promoters and that CREB is reduced in the obese heart [35]. Thus, PKD activation with a consequent decrease in CREB could be a molecular mechanism for abnormal repolarization. It is notable that PKD has a well-described role in the cardiac response to oxidative stress and regulation of metabolism, reviewed in: [58]. In vitro work has shown that PKD activation increases mitochondrial fission, which promotes increased production of mitochondrial ROS [59] it is unclear if this pathologic mechanism applies to the obese heart.

Dysfunctional mitochondria may also have reduced ability to use fatty acids as a fuel source, which could result in myocardial lipid accumulation, worsening the effects of lipid overload in a feed-forward manner [60,61]. The impact of impaired mitochondrial dynamics on ROS production is confirmed by the phenotypic rescue of cardiac myocytes of mice fed an HFD following treatment with mitochondrial fission inhibitors and fusion promoters, enhancing mitophagy and mitochondrial function [62]. Interestingly, recent work indicates that increasing fatty acid oxidation in the heart during HFD may actually be protective with regards to contractile function. This work showed that HFD suppressed mitophagy, and deletion of acetyl-CoA carboxylase 2 (ACC2)—which inhibits mitochondrial import of fatty acids—improved mitophagy and prevented cellular hypertrophy and a decrease in ejection fraction, which was seen in control mice after 24 weeks of high-fat diet-induced obesity [63]. This indicates that mitochondrial consumption of fatty acids is beneficial, implying that excess accumulation or storage of fatty acids in cardiomyocytes is harmful. Cardiac myocytes can store fatty acids in internal lipid droplets, but relatively little is known about the possible pathologic consequences [64].

## 5. De Novo Lipogenesis

Dietary fat is not the only dietary compound that leads to the macro- and microscopic accumulation of fat. Excessive long-term carbohydrate intake can also cause obesity. Fructose, while an isomer of glucose, is metabolized quite differently compared to other simple sugars [65]. Both human and rodent studies show that a fructose-rich diet ((FRD), as opposed to HFD) results in nearly similar myocardial and systemic changes as are seen following an HFD [66,67,68,69,70,71,72]. Excessive consumption of dietary sugar—particularly from sugar-sweetened beverages (e.g., nondiet soda pop)—increases a person’s risk of developing type 2 diabetes mellitus (T2DM) and CVD due to fructose’s metabolism into fatty acid precursor molecules [68,70,71]. Under normal, controlled conditions of carbohydrate (i.e., glucose) metabolism, sugar bypasses insulin-sensitive hepatocytes to enter systemic circulation for delivery to extrahepatic organs. Fructose, however, is metabolized by a kinase unresponsive to hepatic ATP or insulin levels [65]. This results in the unregulated accumulation of phosphorylated fructose (which is trapped in the liver), leading to downstream de novo lipogenesis [71]. This well-established mechanism represents a “fat-free” pathway toward the development of dyslipidemia, hypertriglyceridemia, and subsequent FAO-dependent oxidative damage. Complicating matters further, a detrimental feed-forward cycle of metabolic stress can result from a fructose diet: Hepatic de novo lipogenesis leads to increased insulin resistance in the liver, further promoting de novo lipogenesis from sugars and the accumulation of fat, and so on [65]. The ensuing stores of hepatic lipids are then shuttled systemically in the form of TAG-rich lipoproteins where they can be delivered to the heart and cause damage from lipid-overload.

Even in the absence of dietary fat and fructose consumption, the human body still maintains a mechanism for de novo lipogenesis. Glucose can be converted into fructose by ketohexokinase (KHK) via the polyol pathway. Mice lacking KHK, and thus unable to convert glucose into fructose, are protected from the insulin resistance, dyslipidemia, and weight gain observed in wild-type mice fed a glucose-rich diet in the absence of fructose [68]. This not only shows the necessity of KHK in a fructose-free pathway for de novo lipogenesis, but also confirms the sufficiency of the polyol pathway to promote lipogenesis in the absence of a high-fructose diet.

## 6. An Overview of Arrhythmia Pathophysiology

Abnormal calcium handling within the cardiac myocytes is central for many types of arrhythmias. The contraction of cardiac myocytes begins with the introduction of external calcium into the cytosol of the cell through the L-type calcium channels (LTCCs), signaling the SR to release more calcium into the cytosol via ryanodine receptor channels (RyRs)—specifically the RyR2 isoform in cardiac tissue [73,74]. These RyRs, the largest known ion channel complex, allow for the downstream calcium-dependent processes that occur throughout the cell, e.g., actin—myosin contraction [75,76]. Other channels, such as the inositol 1,4,5-trisphosphate receptor (IP_3_R), also respond to cellular cues to release calcium from the SR [77].

Whereas the release of calcium from the SR marks the initiation of contraction in cardiac myocytes, the removal of calcium from the cytosol following systole is equally vital. The translocation of cytosolic calcium back into the SR by the sarco/endoplasmic reticulum calcium ATPase (SERCA) is essential in diastole and represents a sensitive window of time in which improper calcium clearance can have significant arrhythmogenic consequences [73]. Mutations or covalent modifications of RyR channels can result in the leakage of calcium across the SR membrane during diastole, which can promoter arrhythmias [76,78]. Premature calcium flux from the SR results in untimely depolarization events known as early afterdepolarizations (EADs) and delayed afterdepolarizations (DADs) [73]. Ventricular arrhythmias can be caused by electrolyte imbalance, abnormal calcium handling, and/or myocardial scarring [79]. It is also worth noting that ischemia and subsequent scarring (commonly observed following infarct or substantial tissue damage through other mechanisms) can lead to a physical barrier (e.g., fibrotic tissue) that impedes depolarizing waves throughout the myocardium, promoting arrhythmia. Although myocardial scarring remains a common substrate for fatal ventricular arrhythmias, metabolic abnormalities may also play a significant role, as discussed below.

## 7. Arrhythmogenic Mechanisms Caused by HFD and/or Obesity

A potential explanation for the increased risk of developing arrhythmias in individuals with obesity is in the altered expression of various ion channels and gap junction proteins within the myocardium, which has been shown in mice fed a high-sugar diet and with HFD-induced obesity [80,81,82,83].

This, in addition to fibrotic remodeling of myocardial tissue, offers an electrophysiological mechanism for obesity-related arrhythmias, though the precise details remain unclear. It is possible that these ion channel modifications and fibrosis could be prevented with mitochondria-targeted antioxidant treatment, once again highlighting the impact of mitochondrial ROS in the development of arrhythmogenesis [80].

Arrhythmias require not only a trigger for initiation but also a subsequent mechanism or substrate for perpetuation. Chronic HFD consumption and downstream excess FAO and metabolic stress, along with chronic low-grade inflammation associated with obesity, can trigger the onset of an arrhythmia by cellular modifications that result in calcium leak within the cardiac myocyte. This substrate can lead to RIRR, as mentioned earlier, and may further cause heterogeneous repolarization of cardiac myocytes, a mechanism of arrhythmic propagation. In fact, we have previously shown via optical mapping that rodents fed a high-saturated-fat diet have significantly greater repolarization heterogeneity and increased action potential duration (APD) than mice fed standard chow [27]. This could contribute to sustained arrhythmias. It is important to acknowledge the differences between electrophysiological abnormalities (such as ion channel or gap junction channel expression) seen in cases of HFD- and obesity-related arrhythmogenesis and the physical hinderance of propagation through mechanisms such as post-myocardial infarct scarring.

## 8. Atrial Arrhythmias

AF is the most frequent sustained arrhythmia seen in clinical practice [84]. Although AF usually starts as shorter episodes of tachycardia (paroxysmal AF), episodes become more frequent with longer duration until many patients are in permanent (chronic) AF. Pharmacology has limited efficacy in treating AF [85]. Invasive procedures such as AF ablation, while effective for some patients, may require multiple procedures that have a risk of serious complications. Thus, new approaches are urgently needed to manage AF.

Although human genetics have shown modest power to predict AF, it is largely a disease of age with a contribution from diet and lifestyle (in the absence of structural heart disease, e.g., abnormal heart valves). Diabetes is a well-recognized risk factor for AF [2]. Obesity is also a risk factor and a clinical study showed that weight loss can prevent AF recurrence [86]. Interestingly, different types of dietary fats may influence risk of AF. A post hoc analysis of a clinical trial of the Mediterranean diet intervention showed that increasing olive oil consumption decreased the risk of AF [87]. Fish consumption is also inversely related to incident AF as this is thought to be mediated by fish oils [88]. Consistent with that observation, another study examined serum metabolites in samples from the Cardiovascular Health cohort and found that higher levels of polyunsaturated fatty acids (typically from seafood) were associated with less new-onset AF [89]. The traditional cholesterol panel does not predict new-onset AF [90]. This underscores the point that, although cardiomyocyte lipid metabolism is probably relevant for AF risk, atherosclerosis is not a major cause of AF. These clinical studies indicate that nutrition and obesity are risk factors for AF in humans.

Additional evidence for the role of cardiac metabolism in promoting AF comes from human atrial tissue samples taken from patients undergoing cardiac surgery. One study found abnormalities in atrial tissue metabolism comparing samples from patients with permanent AF to patients who had never had AF [91]. Specifically, the patients who had AF had increased ketones and significant differences in levels of several amino acids compared to patients who were in sinus rhythm. The same study showed that atrial tissue metabolite profiles predicted post-operative AF with 80% accuracy. Post-operative AF may not have the same pathophysiology as AF that occurs spontaneously, but this is proof in principle that cardiac metabolic abnormalities precede AF, and, thus, could promote AF. A group at the Cleveland Clinic used human atrial tissue for messenger RNA microarrays and found that patients with a history of AF, but in sinus rhythm at the time of surgery, had significant decreases in p53 and CREB target genes [92]. CREB has an important role in regulating both metabolism [93] and cardiac ion channels [35,94] and, thus, is a plausible molecular link between metabolism and arrhythmia. Although p53 is best known as a tumor suppressor, it also regulates mitochondrial metabolism [95] and cardiac hypertrophy remodeling [96]. These data from human samples are consistent with the hypothesis that abnormal metabolic regulation could have a role in AF, but the cause and effect are unclear. One plausible link between metabolism and arrhythmia is oxidative stress. A study using human atrial samples showed that NOX2 is activated during AF, resulting in increased ROS [97]. As described above, we have shown that saturated fat, but not unsaturated fat, activates NOX2 in ventricular myocytes, which increases ROS. This, in turn, causes SR calcium leak and mitochondrial dysfunction [19].

Other studies using mice with mutated RyR2 channels, causing increased calcium leak, indicate that mitochondrial oxidative stress may have a role in atrial arrhythmia pathophysiology [98]. Large animal models have been used to investigate the role of abnormal metabolism due to obesity in causing arrhythmia. Obese sheep developed increased epicardial fat and increased atrial fibrosis, which was associated with fractionated electrograms and increased inducibility of AF [99]. Interestingly, the converse may also be true: Atrial arrhythmias may cause abnormal metabolism. Large animal models demonstrate that inducing AF by rapid burst pacing can cause acute derangements in atrial metabolism, with increased oxygen demand and increased lactate production [100]. In a pig model of burst pacing-induced AF, atrial oxygen extraction and lactate production increased during acute AF compared to sinus rhythm [101]. This was interpreted as atrial oxygen demand increasing more than oxygen supply, causing a supply—demand mismatch due to the rapid electrical activation during AF. A more detailed understanding of atrial metabolism during AF could yield novel insights for therapeutic targets.

Recent work has shown that DIO mice have more inducible AF than control mice [80]. Investigating the molecular mechanisms of arrhythmia, the authors found that there was activation of the PKC isoform and NOX2 in the atria. DIO atrial myocytes had increased markers of oxidative stress. Cardiac sodium channel protein levels and current were reduced in DIO atrial myocytes in addition to alterations in LTCC current and Kv channel current. DIO atrial myocytes had shorter average action potential duration compared to control. The DIO atria also exhibited increased fibrosis. Most of these abnormalities were improved by treatment with mitoTEMPO. This work highlights the fact that the electrophysiologic response to obesity is complex, and that reducing mitochondrial ROS via pharmacological antioxidant administration is beneficial.

## 9. Ventricular Arrhythmias

Although it is widely reported that the long-term consumption of a diet high in saturated fat results in increased oxidative stress in various tissues, we do not have a good understanding of how this relates to ventricular arrhythmias. Cardiac lipid overload caused by transgenic over-expression of peroxisome proliferator activated receptor gamma (PPARγ), without obesity or diabetes, causes spontaneous ventricular tachycardia and SCD with prolonged repolarization due to a decrease in Kv channel expression [102]. Several other transgenic models of cardiac lipid overload have been developed, such as the PPARα overexpression mouse and the fatty acid transport protein overexpression mouse [103,104,105]. These transgenic mice consistently have abnormal cardiac ion channel expression, supporting the link between cardiac lipid metabolism and arrhythmogenic remodeling. Although transgenic models are not necessarily physiologic, this is proof in principle that abnormal cardiomyocyte lipid content causes arrhythmia. Our laboratory recently showed that a short duration of high-saturated-fat diet results in the promotion of arrhythmias in a NOX2-dependent manner in mice [27]. As noted above, PKC—activated following exposure to a diet high in saturated fat—subsequently activates NOX2 in the heart [19]. A high-saturated-fat diet results in both oxidation and increased phosphorylation of RyR2, causing increased calcium leak, measured as calcium sparks in isolated cardiomyocytes. As a control, a high-fat diet composed of olive oil, which is mostly composed of oleate, did not cause arrhythmias. Thus, the type of dietary fat is critical for the pathophysiology; not all high-fat diets have the same effects on heart rhythm. Importantly, these results represent the effects of an HFD independent of obesity and other HFD-related conditions—a common and sometimes unavoidable covariate when investigating the effects of an HFD in humans [27,106,107]. After four weeks of HFD, the mice were not obese, had normal ejection fractions by echocardiography, and cardiac histology indicated that there was no increase in fibrosis or hypertrophy [27]. This is important since it demonstrates that metabolic abnormalities can promote arrhythmias in structurally normal hearts. Optical mapping showed increased repolarization dispersion in the ventricles of wild-type (WT) mice after HFD, but not in NOX2 knock-out (KO) mice. Further, WT hearts had significantly more inducible ventricular tachycardia than NOX2 KO hearts after HFD. Part of the arrhythmic effect of NOX2 is due to activation of CaMKII, which phosphorylates the RyR2 channels [108,109]. This causes increased calcium leakage from the SR, which worsens mitochondrial function and promotes arrhythmias [27,77].

Both ROS production and mitochondrial function are important in evaluating the arrhythmogenic effects of an HFD. The pathology likely involves oxidation and phosphorylation of the RyR2 calcium-release channels. Whereas ROS production affects calcium handling through protein oxidation and activating kinases’ pathways leading to ectopic beats, impaired mitochondrial biogenesis and quality control could result in inefficient ATP production from FAO, which could also contribute to abnormal heart rhythm [110]. Though abnormal calcium handling is probably the trigger for arrhythmias, HFD also causes abnormal repolarization, which is a substrate for sustained arrhythmias.

## 10. Additional Metabolic Mechanisms that Potentially Regulate Heart Rhythm

### 10.1. Fructose

As mentioned earlier, the process of arrhythmogenesis following a high-fructose diet is mechanistically similar to that seen following an HFD, resulting in near identical cellular modifications: Oxidation of CaMKII, phosphorylation of RyR2, and increased production of ROS to name a few [66,67,72]. Pharmacological inhibition of CaMKII results in protection against AF in both HFD- and FRD-fed rats, confirming the mechanistic similarity [66,67]. The transcription factor sterol regulatory element-binding protein-1 (SRBP-1) is also upregulated following a high-fructose diet, resulting in the production of lipogenic enzymes as well as the inhibition of lipolytic enzymes [69]. In the presence of a high-fructose diet lacking high-fat content, SREBP-1 signals for the synthesis of fatty acids while the liver is concurrently producing fat via de novo lipogenesis. PPARα, a promotor of FAO, is likewise reduced following a high-fructose diet. Treatment with the PPARα agonist fenofibrate results in restored TAG metabolism, FAO enzyme synthesis, and improved insulin resistance in cardiomyocytes of high-fructose-diet-fed rats [69].

### 10.2. Inflammation

Similar to the effects of a chronic HFD in individuals without comorbidities, abnormal lipid accumulation and systemic low-grade inflammation are observed in those with obesity and diabetes and may contribute to the development of arrhythmias [111]. Levels of the anti-inflammatory cytokine interleukin10 (IL-10) are diminished in animals with obesity, further promoting pro-arrhythmic remodeling [111]). Insulin resistance, a hallmark of T2DM, may cause atrial remodeling and altered expression of calcium channels [66]. Specific to insulin resistance observed in T2DM is the involvement of the NLR family pyrin domain containing 3 (NLRP3) inflammasome, which activates inflammatory cytokines IL-1β and IL-18 and can lead to more severe insulin resistance [112].

### 10.3. Insulin Pathways

There is also evidence the insulin receptor pathway can modulate ion channel function. One group showed that decreased phosphoinositide 3-kinase (PI3K) activity, which is downstream of the insulin receptor, contributes to increased action potential duration in both type 1 and type 2 diabetics’ cardiomyocytes, and this was mediated by increased late sodium current [113]. Another group has shown that type 1 diabetic atrial myocytes have decreased sodium flux, which is corrected by insulin treatment [114]. Although the pathophysiology of type 1 diabetes is different in several important ways from type 2 diabetes, which is much more common in obese adults, the results may have some relevance to DIO and HFD arrhythmogenesis.

### 10.4. Mitochondrial DNA Damage

In a genetically engineered mouse model with accelerated accumulation of mitochondrial DNA damage, older mice exhibited a mosaic pattern of mitochondrial damage in the heart. This heterogeneity resulted in a significant increase in ventricular arrhythmias at both 12 and 18 months [115]. Although this was not an obesity model, it is additional evidence that mitochondrial dysfunction can cause arrhythmias, and the gradual accumulation of mitochondrial DNA damage in the human heart could contribute to the increased frequency of arrhythmias in older people. It is plausible that HFD and/or obesity could significantly exacerbate this pathologic process.

### 10.5. AMPK (Adenosine Monophosphate-Activated Protein Kinase)

Adenosine monophosphate-activated protein kinase (AMPK) is a conserved sensor of cellular energy state. The biology of AMPK has been investigated extensively in the context of ischemia, heart failure, and hypertrophy, but much less is known about the role of AMPK in arrhythmias. AMPK signaling is complex, with both transcriptional and nontranscriptional functions. Activation of AMPK increases glucose utilization, increases mitochondrial biogenesis, and reduces oxidative stress [116]. AMPK is upregulated in the heart in a canine model after one week of AF but downregulated in human samples from patients with chronic AF [117]. It is possible that the early-stage activation of AMPK is compensatory, and then the downregulation at the chronic stage promotes progression of AF. Furthermore, cardiac-specific knock-out of liver kinase B1 (LKB1), which is an activator of AMPK, results in AF in mice [118]. Thus, stimulating AMPK to modulate cardiac metabolism could be beneficial for atrial myocytes in the context of AF and/or HFD. There are reports that AMPK can regulate Kv channels [119], LTCCs [117], and sodium channels [120], but some of these projects used cell lines or acute pharmacologic treatment of isolated ventricular myocytes, which may not correlate with long-term in vivo effects. It would be interesting to evaluate these currents in isolated atrial myocytes after long-term activation of AMPK in vivo. Concerningly, although in vivo pharmacologic AMPK activation improved glucose homeostasis in rodents and monkeys, it induced cardiac hypertrophy [121]. This hypertrophy did not appear pathologic in these animal models but could represent a limitation for long-term use of AMPK activators in humans.

### 10.6. Adiponectin and Other Adipokines

Adiponectin is an insulin-sensitizing adipocytokine and is thought to have cardioprotective effects [122]. Serum levels are decreased in obesity but increased in heart failure [123]. Increased epicardial adiponectin levels predict less post-operative AF in patients [124]. However, in the Framingham study, serum adiponectin levels did not predict incident AF [125].

There are conflicting data regarding insulin resistance in adiponectin KO mice [126,127]. Whole-body adiponectin overexpression mice have less fat accumulation and improved lifespan on HFD [128]. Adapter protein adapter protein containing PH domain, PTB domain and leucine zipper motif 1 (APPL1) is downstream of adiponectin, and APPL1 overexpression mice have less cardiac lipid and do not develop systolic dysfunction on HFD after 16 weeks [129]. The signal transduction pathways downstream of adiponectin appear to have several branches, but the beneficial effects in the heart may be mediated by AMPK [130]. In the liver, adiponectin decreases activity of SREBP1, which regulates enzymes involved in fatty acid synthesis [131]. Adiponectin KO mice exhibit larger myocardial infarct after surgical coronary ligation compared to control mice, in part mediated by increased tumor necrosis factor (TNFα) and decreased activation of AMPK [132]. Adiponectin may have direct electrophysiologic effects. In humans, it has an independent inverse relationship with corrected QT interval QTc [133]. In the central nervous system, adiponectin is known to regulate potassium channels [134]. It is unclear if this is also true in cardiac myocytes. Isolated cardiac myocytes from adiponectin KO mice have calcium transients of longer duration, similar to heart failure myocytes; this is explained by the finding that adiponectin enhances SERCA2 activity via CaMK-phospholamban signaling pathway [135].

Other adipokines may also have electrophysiologic effects. Leptin (a hormone stored and secreted from adipocytes) has also been shown to be important in the development of HFD-dependent atrial fibrosis and AF, which may relate to the importance of fat loss for arrhythmia protection in overweight individuals [136].

## 11. Dietary and Pharmacological Interventions

### 11.1. Dietary Modification

It seems likely that the most impactful dietary/lifestyle modifications for reducing the risk of arrhythmias include consuming foods low in saturated and trans fats and maintaining a healthy BMI. Cellular alterations resulting from obesity—such as tissue fibrosis, atrial remodeling, and diminished conduction velocity—are reversible with weight loss [25]. The consequences of obesity-derived lipotoxicity and oxidative damage are also attenuated upon reaching a healthy BMI and proper management of T2DM [25]. As recently discussed, avoiding dietary saturated and trans fats is not sufficient to confer protection against pro-arrhythmic metabolic stress. It is equally important to monitor sugar consumption, particularly fructose, as excess carbohydrate intake also results in increased FAO and lipid accumulation that can lead to oxidative cellular damage. For reference, the recommended daily intake for carbohydrates (set by the Food and Nutrition Board and Institute of Medicine) is 130 g per day. One 20 oz bottle of soda contains roughly 60 g of sugar, half of which is fructose alone.

### 11.2. Antioxidant and Pharmacological Remedies

A significant potential benefit in the utilization of antioxidants as anti-arrhythmic therapy is the lack of pro-arrhythmic side effects sometimes observed in traditional pharmacological interventions, particularly due to altered APD [137]. Although clinical trials of antioxidants for cardiovascular disease have been largely disappointing, there is some clinical evidence supporting an anti-arrhythmic effect [138,139]. It may be the case that antioxidants are only beneficial in certain disease states, where increased ROS have a central role in the pathophysiology, or that more potent and specific antioxidants are required to reach a therapeutic concentration. Many possibilities are emerging with the goal of correcting diet- and/or obesity-induced oxidative damage. Several publications with in vivo data are summarized in Table 1. As discussed earlier, NOX2 inhibition prevents the pro-arrhythmic effects of an HFD by halting the production of ROS [27]. Also discussed above is the potential for IL-10 therapy, inhibiting the pathology of atrial remodeling and fibrillation through its antioxidant properties [111].

Another therapeutic target is the mitochondrially targeted antioxidant mitoTEMPO. The specificity of this mechanism allows for the prevention of mitochondrial ROS-induced proteomic alterations that follow an HFD, providing a feasible option for reducing risk of HF and SCD [110,140]. In animal models, mitoTEMPO reduces premature ventricular contractions that result from abnormal calcium handling and mitochondrial ROS-induced oxidative stress [54]. The organic alcohol farnesol has also recently been shown to exert cardioprotective effects against arrhythmias [141]. However, its function is not strictly antioxidant as it primarily acts as an LTCC inhibitor and protein geranylgeranylator [142]. It is, thus, essential to continue delineating the mechanism of anti-arrhythmic antioxidants without underlying mechanistic alterations.

As mentioned previously, activation of PKD and CaMK appears to be important for the pathophysiology of the obese and/or HFD heart. Chemical inhibitors of these kinases could be effective; however, off-target effects could result in unanticipated consequences. Activation of AMPK could be beneficial for heart rhythm in the context of obesity and/or HFD but, to the best of our knowledge, this has not been tested. Though this discussion of potential pharmacological targets is certainly not exhaustive, a final therapeutic intervention involves c-Jun N-terminal kinase, or JNK. This kinase, responsible for the activation of CaMKII, is a factor in the development of atrial arrhythmias [143]. Left unchecked, this pathway results in the disturbance of diastolic calcium handling, as mentioned earlier in the review [143,144]. Targeted inhibition of JNK could be yet another mechanism for combatting the oxidative stress and pro-arrhythmogenic effects of a chronic HFD.

## 12. Conclusions and Future Directions

**Figure 1:**
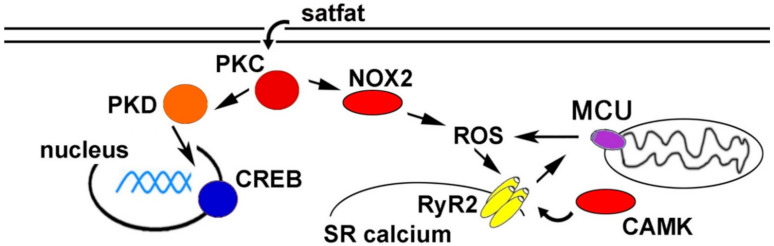
Schematic of proposed pathways promoting arrhythmia during high fat diet (HFD) and/or obesity. CAMK = calcium/calmodulin-dependent protein kinase, CREB = cAMP response element-binding protein, MCU = mitochondrial calcium uniporter, NOX2 = NADPH oxidase 2, PKC = protein kinase C, PKD = protein kinase D, ROS = reactive oxygen species, RyR2 = ryanodine receptor 2, SR = sarcoplasmic reticulum.

Cardiac arrhythmias are responsible for many cardiovascular disease-related deaths worldwide. While arrhythmia pathogenesis is complex and multi-factorial, there is increasing evidence for metabolic causes. Obesity, diabetes, and chronically consuming high-fat foods significantly increase the likelihood of developing arrhythmias. Although these correlations are well established, mechanistic explanations connecting an HFD to arrhythmogenesis are incomplete. Animal models have been used to better understand the molecular mechanisms. A working model is presented as a schematic in Figure 1. These studies have indicated several potential therapeutic targets to prevent arrhythmias caused by obesity and/or HFD.

More research is needed to better gain an understanding of the mechanisms that connect abnormal metabolism and suboptimal nutrition to arrhythmias. Mitochondrial dysfunction appears to be critical and more information is emerging. As it stands, there is very little work investigating the role of mitophagy in arrhythmias. There is some debate as to the role of mitochondrial calcium in arrhythmias, which is regulated (in part) by the mitochondrial calcium uniporter (MCU) [145]. The mechanisms that link obesity to cardiac fibrosis also lack clarity and could be a promising area for future investigation. The potential pathologic role of intracellular lipid droplets is also relatively under-studied. Additionally, sex differences in cardiac response to obesity have been detected in human studies, calling for animal models to better understand these differences.

## Figures and Tables

**Table 1:** Antioxidants preventing arrhythmia in vivo.

	Dey et al.	Joseph et al. ‘16	Joseph et al. ‘19	McCauley et al.	Sanchez et al.
**Species**	Guinea pig	Mouse	Mouse	Mouse	Mouse
**Disease Model**	Heart failure	Transgenic-induced cardiac lipid overload	DIO	DIO	DIO
**HFD**	N/A	N/A	60% fat, palm or olive oil	60% fat, lard	60% fat, lard
**Diet Duration**	N/A	N/A	4 weeks	~5 months	8 weeks
**Antioxidant**	mitoTEMPO	mitoTEMPO	Apocynin	mitoTEMPO	Apocynin
**Results**	Prevented HF, decreased vent. arrhythmia	Decreased PVCs	Prevented PVCs	Reduced AF, restored Na_v_1.5, K_v_1.5 expression	Prevented vent. arrhythmia
**Reference**	90	51	24	72	89
